# A Testimonial

**Published:** 1905-11

**Authors:** 


### A Testimonial.

I thought that my health was as good as the next,
  But I learned that it was terribly bad;
For I found, after reading the newspaper text
  Of a loud patent medicine ad.,
That mushrooms were growing all over my liver,
  That something was loose in my heart,
That due to my spleen all my nerves had turned green
  And my lungs were not doing their part.
I wrote Dr. Sharko and got as an answer,
"The wart on your thumb is incipient cancer."

I've taken Ze-ru-na for forty-nine days,
  And Scamp Bark, my symptoms to gag;
And isn't it queer—all my pains disappear
  When the medicine gives me a jag?
A "lovely sensation" I got from them all,
  Which banishes carking annoy.
So gaily I drink 'em—and Lydia Pinkum.

  To give all poor sufferers hope.
Much pain I've endured, but I'm "positive cured"
  So long as I'm taking the dope.
The baby has spasms, my wife's throwing fits,
  And I'm feeling fuzzy and bad—
For I feel we've amassed all the symptoms at last
  Which you read in the medicine ad.
The ready-made cure and the angels who make it
Thus comfort and bless the poor devils who take it!

—*Wallace Irwin in Collier's Weekly.*

---

A RARE OPPORTUNITY is offered to secure a $2500 practice and a beautiful home and small farm in a middle Texas county, in the richest farming and cattle section of the State. The property consists of well improved 60 acres in a thriving village, 30 in cultivation and 30 divided in two pastures of fine Bermuda grass, a nice five-room cottage, with bath and all other conveniences, large barn and other outhouses, abundant water, well and windmill and private waterworks. Forty-eight acres will be sold separately if desired, and the 12 acres with the improvements is offered for $2000, or the whole for $3500. This is a real bargain and a rare opportunity to step into a paying practice in a splendid section of country and a prompt paying community. Terms on request. Dr. C. F. C., care this Journal, Austin, Texas.

---

A SUCCESSFUL medical practitioner of many years' standing makes the following statement: "There are a large majority of combinations which extemporaneous pharmacy can not prepare properly; and I know that through the dishonesty, ignorance, or indifference of many retail druggists we are not able to get on prescriptions the very best drugs; hence it is to the manufacturing pharmacist, whose best interest lies in the purity and uniformity of his product, that we must look for our most reliable remedies. I endorse worthy proprietaries, but I most heartily condemn the great tendency of the 'half-baked,' so-called manufacturing 'chemist,' to foist upon the profession and public cheap imitations of standard preparations."

